# Total Hip Arthroplasty After Femoral Neck Fracture in an Ipsilateral High Transfemoral Amputee With Less Than 50% Femoral Length

**DOI:** 10.7759/cureus.95722

**Published:** 2025-10-30

**Authors:** Tomasz Pobozy, Kacper Janowski, Klaudia Michalak, Natalia Rulewska, Filip Grabowski, Wojciech Konarski

**Affiliations:** 1 Department of Orthopaedics, Medicover Hospital, Warsaw, POL; 2 Department of Internal Medicine, Specialist Regional Hospital in Ciechanów, Ciechanow, POL; 3 Department of Orthopaedics, Medical Rehabilitation Center, Legionowo, POL

**Keywords:** femoral neck, porous acetabular cup, tha, total hip arthroplasty, transfemoral amputation

## Abstract

Total hip arthroplasty (THA) in ipsilateral transfemoral amputees is rare and technically demanding; literature seldom quantifies stump length. We report a 69-year-old male with ipsilateral high transfemoral amputation (<50% femoral length) who sustained a displaced subcapital femoral neck fracture and underwent primary THA. The residual femoral stump measured approximately 215 mm (46.7% of contralateral femoral length) on preoperative CT-based templating. Perioperative management addressed positioning, manipulation of the short limb, and component alignment. At eight months, the patient ambulated with a prosthesis and crutches at the pre-injury functional level, reported no hip pain or instability, and radiographs confirmed stable fixation. This case adds a quantified example of high transfemoral amputation with documented mid-term recovery and practical recommendations for exposure, manipulation, and component version planning. Quantifying stump length and tailoring intraoperative technique are key to safe THA in high transfemoral amputees; favorable outcomes can be achieved with individualized planning.

## Introduction

Femoral neck fractures represent nearly half of all hip fractures in the elderly, carrying high morbidity and mortality [1,2]. Management is further complicated in amputees due to altered biomechanics, unloading-related bone demineralization, and increased fall risk [3-5]. Ipsilateral transfemoral amputees pose additional challenges, including exposure, limb manipulation, and alignment, alongside higher rehabilitation demands [6,7]. 

Although arthroplasty is generally preferred over internal fixation in elderly patients with poor bone quality [2,8], evidence in amputees is limited to isolated reports and small series. A recent narrative review summarized 39 cases (13 above-knee, 23 below-knee, three through-knee), but quantitative data on stump length were seldom included [6]. 

We report a case of femoral neck fracture in a patient with an ipsilateral high transfemoral amputation (<50% femoral length) treated with THA and eight-month follow-up, emphasizing the surgical relevance of stump quantification, intraoperative manipulation, and tailored rehabilitation.

## Case presentation

**Figure 1:**
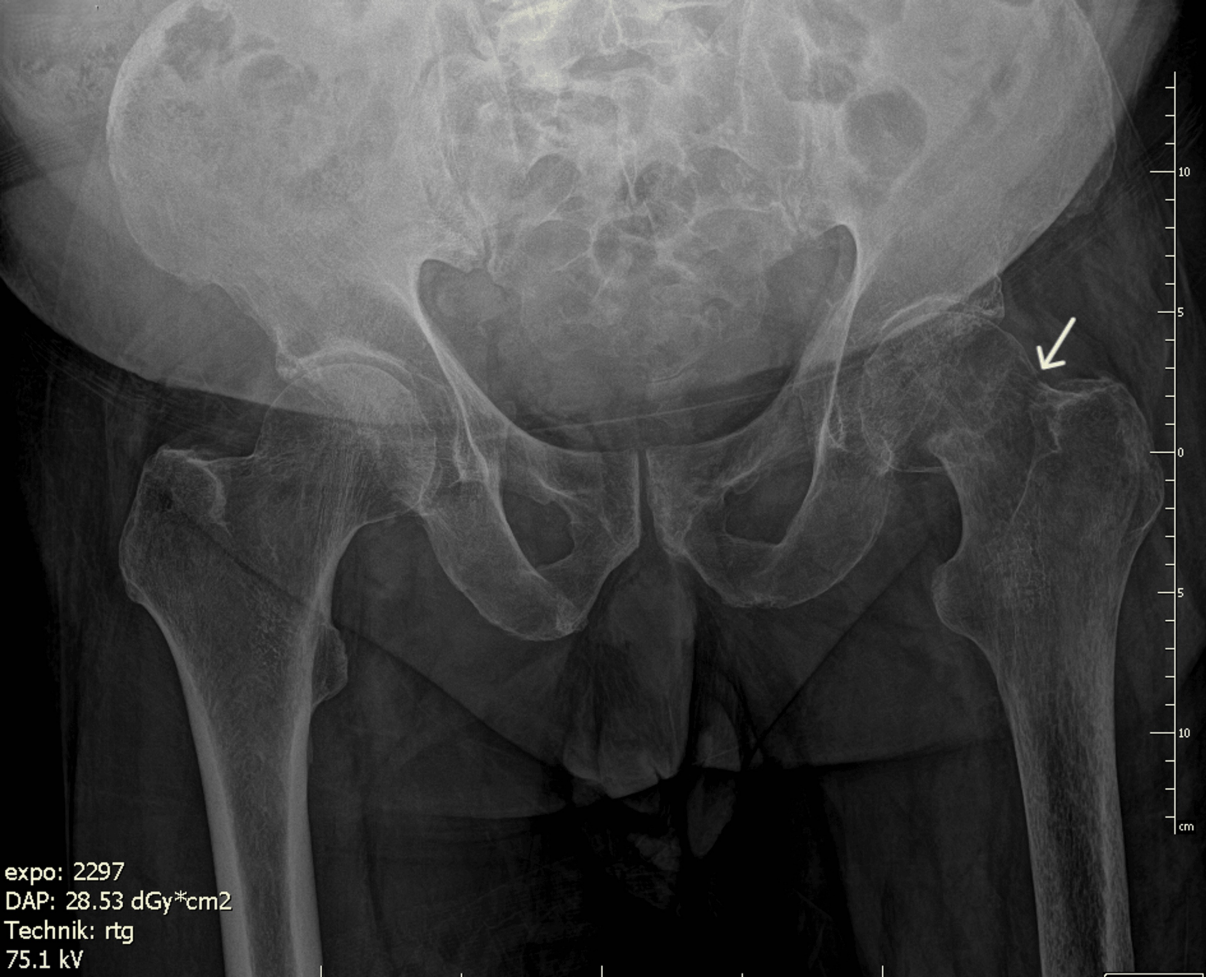
Preoperative AP radiograph showing displaced subcapital femoral neck fracture. The white arrow indicates a subcapital fracture of the femoral neck.

**Figure 2:**
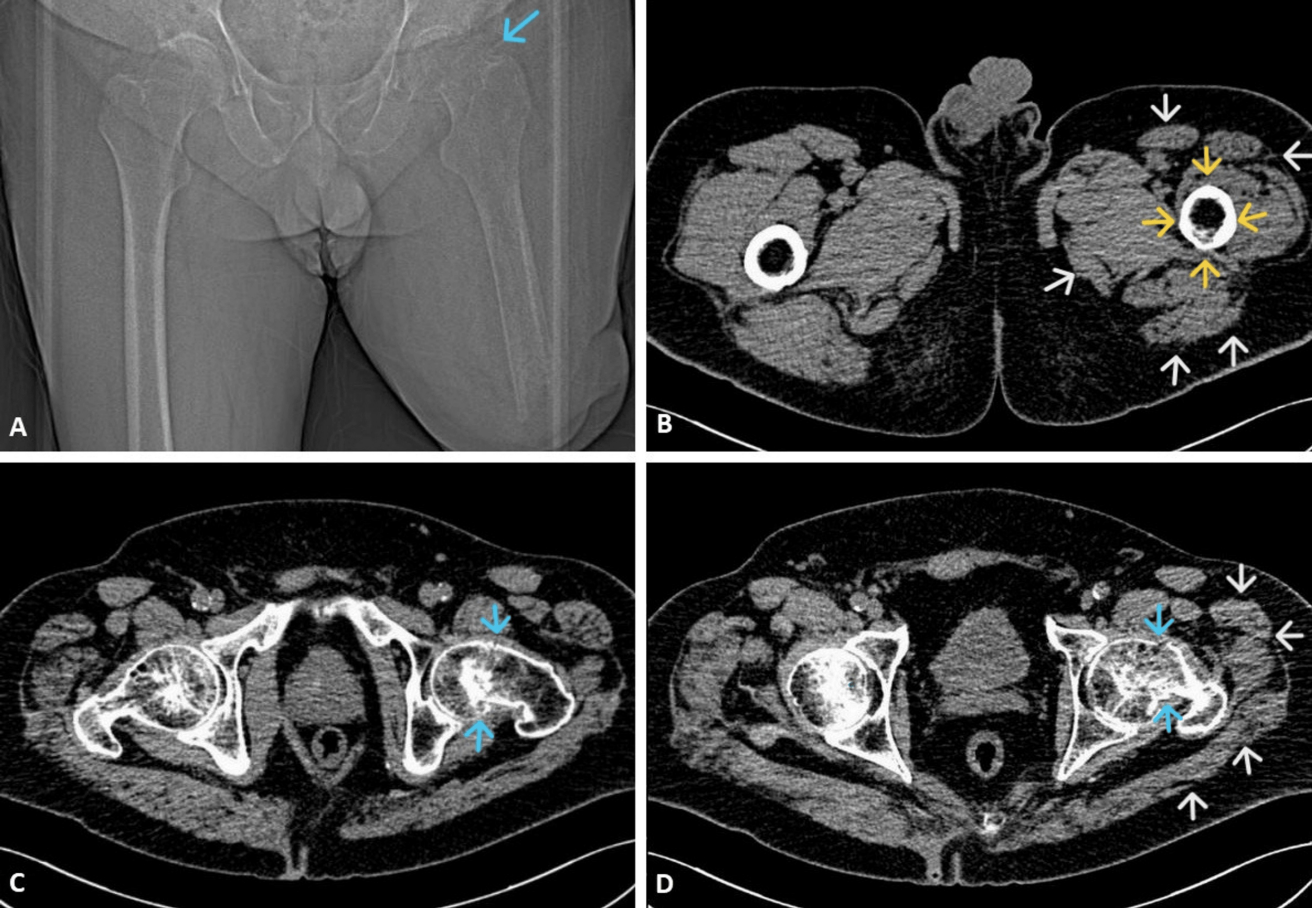
Preoperative CT imaging. A - coronal scout view demonstrating displaced subcapital femoral neck fracture of the left hip; B - axial CT slice showing cortical thinning of the femoral shaft (yellow arrows) and marked muscle atrophy (white arrows); C - axial CT slice highlighting the fracture line (blue arrows); D - axial CT slice showing both muscle atrophy (white arrows) and the fracture line (blue arrows).

A 69-year-old man with a left transfemoral amputation presented after a fall with acute hip pain and inability to mobilize. Before injury, he ambulated independently using a prosthesis and crutches. Radiographs and CT confirmed a displaced high subcapital femoral neck fracture (Figure 1, Figure 2).

The residual femoral stump measured 215 mm, corresponding to 46.7% of the contralateral femoral length (~460 mm measured from the greater trochanter to the knee joint line). Comorbidities included ischemic heart disease (percutaneous coronary intervention (PCI) and minimally invasive direct coronary artery bypass (MIDCAB)), heart failure, hypertension, chronic kidney disease stage G3a, benign prostatic hyperplasia, right knee osteoarthritis, and minor contralateral foot amputation.

Given the patient’s pre-injury independence and the poor prognosis of fixation in osteoporotic bone, primary arthroplasty was indicated.

Operative details

Under spinal anesthesia via a posterior approach, two temporary threaded pins connected by a rod were inserted into the residual femur to facilitate manipulation and exposure. A porous titanium acetabular shell with adjunctive screw fixation and a dual-mobility articulation was implanted. The cementless tapered-wedge femoral stem (neck-shaft angle 132°) achieved stable metaphyseal fixation; a metal 28 mm head (medium offset) was used.

Anteversion of native acetabulum: ~16°, increased intraoperatively by ~5° → final cup anteversion ≈ 21°. 

Femoral stem anteversion: ~15-20°, resulting in a combined anteversion ≈ 36-41°. 

Offset restoration: Within ±5 mm of the contralateral side. 

Leg-length discrepancy (LLD): +2-3 mm, clinically insignificant. 

**Figure 3:**
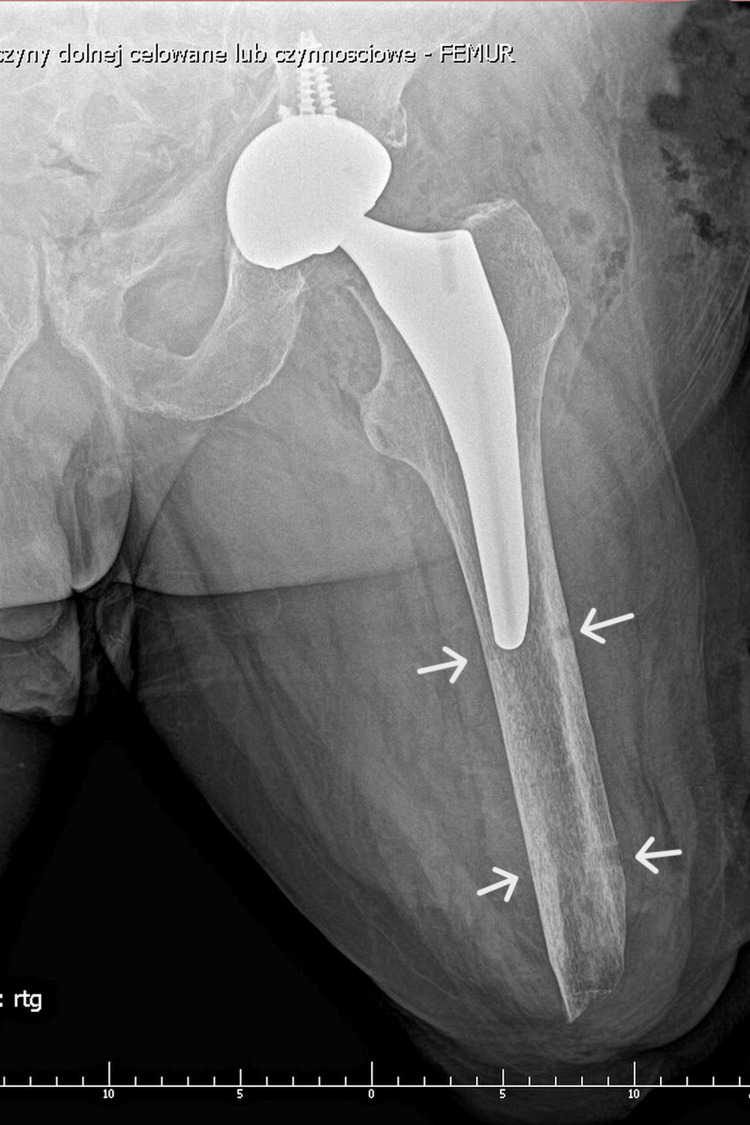
Immediate postoperative AP radiograph. Immediate postoperative AP radiograph showing stable acetabular and femoral components. Arrows indicate the sites where temporary rods were inserted into the residual femur to facilitate intraoperative manipulation.

**Figure 4:**
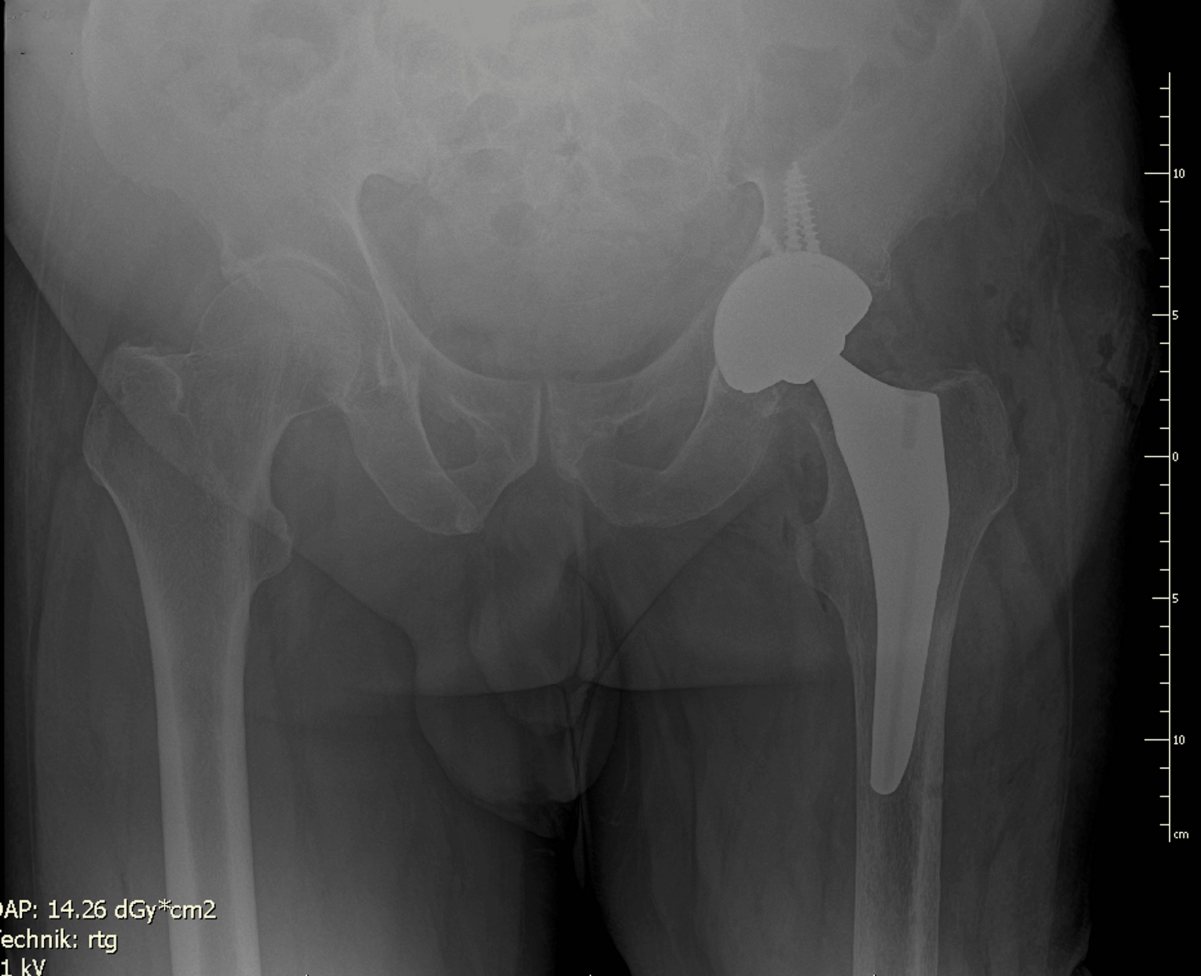
Immediate postoperative AP radiograph showing stable acetabular and femoral components.

Local intra-wound antibiotic prophylaxis (gentamicin + vancomycin) complemented intravenous cefazolin. Postoperative AP pelvis radiograph confirmed stable cup and stem alignment (Figures 3, 4). 

Postoperative course

Full weight bearing as tolerated (WBAT) was initiated from postoperative day 1 [8]. The patient completed a four-week inpatient rehabilitation program tailored for transfemoral prosthesis users. 

Week 0-1: Early mobilization (24 h), isometric exercises (quadriceps, gluteal), hip range of motion (ROM) ≤90° flexion, abduction ≤30°, prosthesis training, and anti-dislocation education. 

Week 2: Gait with two crutches, stair practice, balance drills (closed-chain), endurance goal ≥200 m (six-minute walk test (6MWT)). 

Week 3: Gait retraining with prosthesis, abductor strengthening, and low-level obstacle walking. 

Week 4: Independent transfers and activities of daily living (ADL)(bed, chair, toilet), 10-20 min continuous walking, home exercise education. 

At eight months, the patient ambulated with a prosthesis and crutches without hip pain or instability, regained pre-injury functional level, and demonstrated the following ROM: flexion 100-110°, extension 0-10°, abduction ~30°, adduction ~20°, internal rotation ~15°, external rotation ~25°. The patient was able to ambulate with a prosthesis and two crutches for a distance of ≥300-500 m at a time, independently rise from a seated position, and ascend a flight of 8-10 steps with minimal assistance for safety.

## Discussion

Femoral neck fracture management in transfemoral amputees remains technically demanding. Internal fixation, although feasible, carries a high risk of nonunion and avascular necrosis in elderly or vascularly compromised patients. Arthroplasty enables early mobilization and faster rehabilitation [9,10]. Dual-mobility articulation can mitigate dislocation risk in amputees with impaired muscular control and altered spinopelvic dynamics [11-15].

A few reports have quantified stump length precisely. Diamond et al. [16] described a 130 mm stump with favorable 2-year outcomes using a short stem. Zhao et al. [17] reported 129 mm via the anterior approach, while Christidis et al. [18] mentioned a “high” amputation without measurement. Our quantified case (~215 mm, 46.7%) thus provides valuable comparative data. Precise stump measurement supports exposure planning and component alignment. Temporary fixation devices (threaded pins + rod) facilitated controlled manipulation of the short femur, improving accuracy [6]. While a cemented stem can reduce the risk of intraoperative fracture in atrophic femora, the present patient exhibited satisfactory metaphyseal bone stock, allowing secure press-fit fixation and preserving future revision options. 

Rehabilitation tailored for prosthesis users is essential - early WBAT, proprioception, and abductor training are key for regaining pre-injury gait symmetry. 

This report also has limitations. It represents a single case, limiting the generalizability of the findings. Patient-Reported Outcome Measures (PROMs) were not collected, and follow-up evaluation focused on mobility with the prosthesis. Nevertheless, the documentation of a quantified high-level amputation and favorable recovery at 8 months provides valuable insight for clinicians facing similar complex scenarios.

## Conclusions

Even in patients with high transfemoral amputation (<50% femoral length), total hip arthroplasty can restore pre-injury function when planning and technique are individualized. Explicit quantification of stump length, attention to combined anteversion, and use of manipulation aids and dual-mobility bearings enhance safety and stability. Importantly, this case highlights that a high-level transfemoral amputation should not be considered a contraindication to THA. With individualized surgical strategy and focused rehabilitation, excellent mid-term results are achievable. Our findings reinforce that documenting stump length and functional outcomes is essential for building stronger evidence in this rare but challenging group of patients.
